# TLR2, CCR1, IRF8, and CCL4 as biomarkers for atherosclerosis progression and therapy response: A multi-omics study

**DOI:** 10.1097/MD.0000000000046647

**Published:** 2025-12-26

**Authors:** Wangwei Zhou, Min Huang, Han Li, Jian Shi

**Affiliations:** a Cardiology Department, Liuzhou People’s Hospital, Liuzhou, Guangxi, China; b Cardiology Department, The People’s Hospital of laibin, Liuzhou, China.

**Keywords:** atherosclerosis, diagnostic biomarkers, diagnostic model, immune infiltration, molecular docking

## Abstract

Atherosclerosis (AS) is a growing vascular disease linked to plaque buildup, causing blood flow issues. Current diagnosis relies on symptoms and imaging, which are limited for early detection and plaque biology assessment. Treatments focus on symptoms but don’t address root causes, leading to complications. This study aims to find new diagnostic markers and therapies using bioinformatics and machine learning. Data from gene expression omnibus datasets (GSE28829 for gene expression, GSE159677 for single-cell analysis) were analyzed via WGCNA to identify gene modules, Limma for differentially expressed genes, and gene ontology/KEGG for pathway enrichment. Protein-Protein Interaction networks, machine learning (least absolute shrinkage and selection operator, Random Forest, artificial neural network), immune infiltration (CIBERSORT), and single-cell RNA-seq were used. A nomogram model was built, and candidate drugs (e.g., simvastatin) were tested via molecular docking. Key modules (turquoise) and 238 differentially expressed genes linked to immune processes. Four biomarkers (toll like receptor 2, CCR1, interferon regulatory factor 8, CCL4) showed high diagnostic accuracy (AUC > 0.8). Immune analysis revealed altered macrophage/T cell profiles, with biomarkers correlating to monocyte/macrophage activity. The nomogram model was robust, and simvastatin docked strongly to target proteins. toll like receptor 2, CCR1, interferon regulatory factor 8, and CCL4 are novel AS biomarkers linked to immune pathways. The nomogram aids risk prediction, and simvastatin shows potential as a targeted therapy. Findings advance AS understanding and offer tools for early diagnosis and personalized treatment.

## 1. Introduction

Atherosclerosis (AS) is a progressive and systemic vascular disease characterized by the accumulation of lipid - rich plaques within the arterial walls, leading to arterial narrowing, reduced blood flow, and potential tissue ischemia.^[1,2]^ Over the past few decades, the global prevalence of AS has witnessed a remarkable increase, driven by factors such as aging populations, rising prevalence of risk factors (e.g., obesity, diabetes, hypertension), and lifestyle changes.^[3]^ As a major contributor to cardiovascular morbidity and mortality, AS not only significantly impairs patients’ quality of life by causing symptoms like intermittent claudication and critical limb ischemia but also exerts a substantial economic burden on healthcare systems and society.^[4]^

Current diagnosis of AS primarily depends on a combination of clinical symptoms, physical examination (e.g, ankle-brachial index measurement), and imaging techniques including duplex ultrasonography, computed tomography angiography (CTA), and magnetic resonance angiography.^[5,6]^ However, these approaches are associated with several limitations. The clinical manifestations of AS, especially in its early stages, can be subtle and non - specific, often leading to delayed diagnosis. Imaging methods, although effective for visualizing arterial stenosis, are costly, time - consuming, and may expose patients to radiation (in the case of CTA).^[7]^ Moreover, they lack the ability to provide insights into the biological activity of atherosclerotic plaques, such as inflammation and vulnerability, which are critical for predicting disease progression and cardiovascular events.^[8]^ In terms of treatment, current strategies mainly focus on risk factor management (e.g, statins for lipid - lowering, antiplatelet agents) and revascularization procedures (e.g, angioplasty, bypass surgery).^[9]^ While these interventions can alleviate symptoms and improve blood flow, they do not address the underlying pathophysiological mechanisms of plaque formation and progression.^[10]^ Additionally, revascularization procedures carry risks of complications, and recurrence of arterial narrowing remains a common issue.^[11]^

Given the challenges in diagnosis and treatment outlined above, there is an urgent need to identify novel and reliable diagnostic biomarkers for AS. These biomarkers hold the potential not only to enhance the accuracy and timeliness of early-stage diagnosis but also to serve as critical entry points for unraveling the underlying molecular mechanisms that drive plaque formation and instability. By exploring the relationship between diagnostic biomarkers and the pathogenesis of the disease, it is anticipated that new therapeutic strategies targeting these biomarkers can be developed, thereby providing more effective and personalized treatment regimens for AS patients. To achieve this objective, a comprehensive analysis was conducted on a large cohort of AS patients. Through the integration of advanced bioinformatics techniques, high-throughput sequencing, and machine learning algorithms, this study successfully screened out 4 key diagnostic biomarkers for AS, namely TLR2, CCR1, IRF8, and CCL4. Additionally, a nomogram diagnostic model with favorable predictive performance was constructed, and simvastatin was identified as a potential therapeutic agent for AS, likely exerting its effects through a multi-target mechanism. Our ultimate goal is to explore the feasibility of developing innovative therapeutic approaches based on these biomarkers. Such advancements could potentially revolutionize the current management of AS and improve the long-term prognosis of affected patients.

## 2. Research approach

### 2.1. Data source

**Figure 1.**
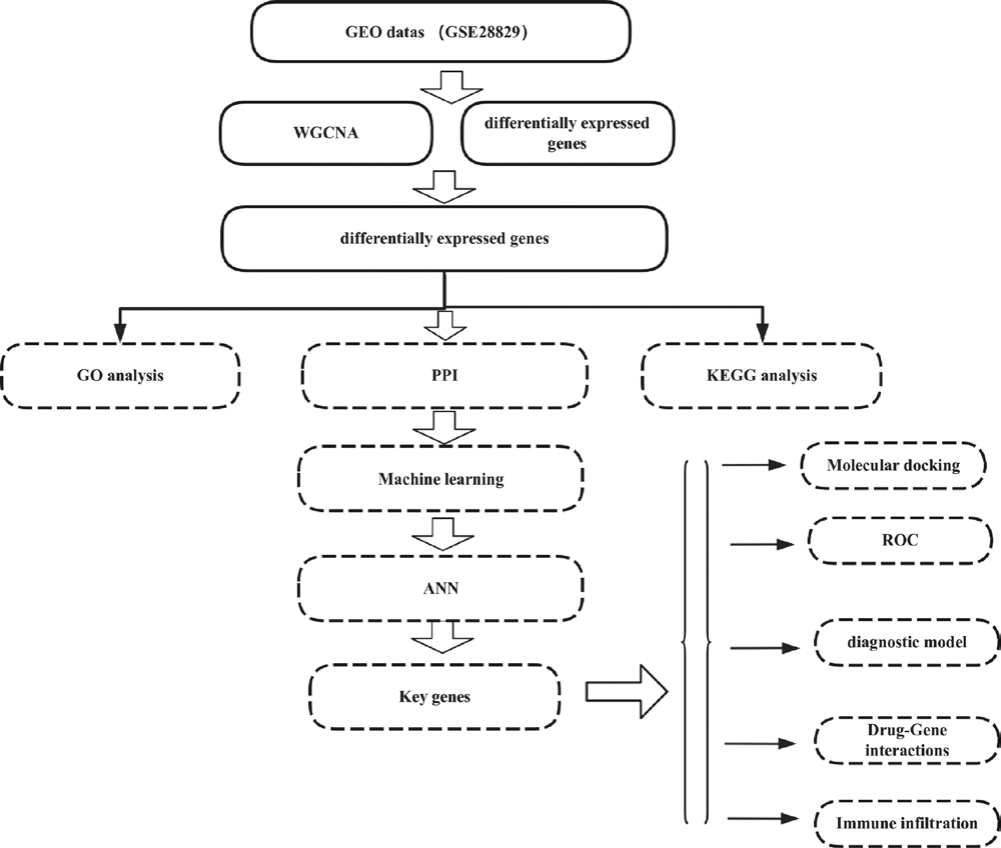
The flowchart of the study.

In this study, all datasets analyzed were derived from the gene expression omnibus database (https://www.ncbi.nlm.nih.gov/geo/).^[12]^ The dataset GSE28829 was used as the experimental group, which included gene expression arrays of 13 early atherosclerotic plaque (EA) samples and 16 advanced atherosclerotic plaque (AA) samples extracted from human carotid arteries. Subsequently, single-cell transcriptome data (GSE159677) were obtained from the gene expression omnibus database. Using R and the Seurat package,^[13]^ we performed a series of analyses, including data preprocessing, quality control, dimensionality reduction, clustering, and cell type annotation. The expression levels of the identified target genes were validated in this dataset. The analytical workflow of this study is illustrated in Figure 1.

### 2.2. WGCNA analysis

To investigate gene modules closely linked to disease clinical characteristics, this study utilized the Weighted Gene Co-expression Network Analysis (WGCNA) package in R for analysis.^[14]^ First, hierarchical clustering analysis was performed to screen samples, efficiently identifying and excluding abnormal outlier samples. Next, the “Pick Soft Threshold” function in the WGCNA package was applied to determine an appropriate soft-threshold parameter β. Using this parameter, a gene adjacency matrix was constructed and subsequently converted into a topological overlap matrix. Leveraging topological overlap matrix metrics, genes with comparable expression profiles were clustered into distinct modules via average-linkage hierarchical clustering. Finally, correlation coefficients between each gene module and disease clinical traits were calculated to elucidate their association strength.^[15]^

### 2.3. Identification of differentially expressed genes

The Limma package was employed to identify differentially expressed genes (DEGs). DEGs were defined by a *P*-value <.05 and an absolute log₂ fold change ≥ 0.585.^[16]^

### 2.4. Functional enrichment analysis

In this study, gene ontology analysis, encompassing 3 domains of biological processes, cellular components, and molecular functions, together with kyoto encyclopedia of genes and genomes pathway enrichment analysis,^[17,18]^ were conducted. Specifically, these analyses were implemented using the ClusterProfiler package in R within the Disease Ontology framework.

### 2.5. PPI network analysis

Protein-protein interaction (PPI) networks for the identified genes were constructed via the STRING tool and visualized using Cytoscape.^[19]^ The MCODE plugin was employed to analyze modules within the PPI networks, whereas the cytoHubba plugin assisted in the identification of hub genes.^[20]^

### 2.6. Machine learning and clinical characteristic analysis

To systematically identify key drug-target genes, we employed advanced machine learning approaches. Feature selection was performed using: least absolute shrinkage and selection operator regression (implemented via the glmnet R package) to enhance model interpretability through L1 regularization^[21]^; Random Forest (RF) analysis (randomForest package) for evaluating feature importance^[22]^; and an Artificial Neural Network (ANN) architecture (neuralnet and neuralnettools packages) to capture nonlinear relationships.^[23]^ Model performance was rigorously assessed using receiver operating characteristic curve analysis.

### 2.7. Immune infiltration and single-cell analysis

We performed comprehensive immune cell quantification using CIBERSORT’s deconvolution algorithm to estimate the relative proportions of 22 distinct immune cell subtypes from bulk transcriptomic data.^[24]^ Subsequent correlation analysis (Pearson method) revealed significant associations between candidate genes and specific immune cell populations. For single-cell resolution analysis, the GSE159677 dataset was processed through an established computational pipeline: initial quality control filtered low-quality cells and potential doublets, followed by principal component analysis of the top 2000 variable genes and UMAP-based nonlinear dimensionality reduction for cluster visualization.^[25]^ Cell populations were rigorously annotated using SingleR’s reference-based classification system, while differential expression analysis identified cluster-specific markers, enabling systematic characterization of cellular heterogeneity within the atherosclerotic microenvironment.

### 2.8. Construction and validation of the diagnostic model

Based on the identified key genes, we developed a diagnostic model to predict the risk of AS. The model was constructed using a dynamic nomogram and decision curve, while calibration curves and clinical impact curves were employed to assess the accuracy of the model’s predictions. Finally, we generated receiver operating characteristic curves using R software to further validate the model’s predictive performance.

### 2.9. Drug–gene interactions and protein–protein docking analyses

This investigation employed a dual-method computational strategy to identify therapeutic candidates and decipher their mechanisms of action. Initially, we leveraged the Drug Signatures Database (DSigDB) to systematically map hub genes to potential therapeutic compounds, utilizing its extensive repository of validated drug-gene interactions.^[26]^ For structural validation, we performed in silico molecular docking studies: drug molecular structures were acquired from PubChem, while corresponding target protein conformations were retrieved from the RCSB Protein Data Bank, prioritizing crystallographically resolved structures. The CB-Dock2 web server facilitated automated binding site prediction and protein-ligand docking simulations, generating critical interaction metrics including binding free energies and intermolecular contact patterns.^[27]^ This integrated approach enabled rigorous evaluation of both pharmacological potential (through DSigDB screening) and structural feasibility (via docking analysis), providing a comprehensive framework for therapeutic development. It should be noted that the molecular docking results in this study are solely based on in vitro computational simulation analysis, without verification by cellular and biochemical experiments; thus, the conclusions have certain speculative nature.

## 3. Results

### 3.1. WGCNA-based module identification

**Figure 2.**
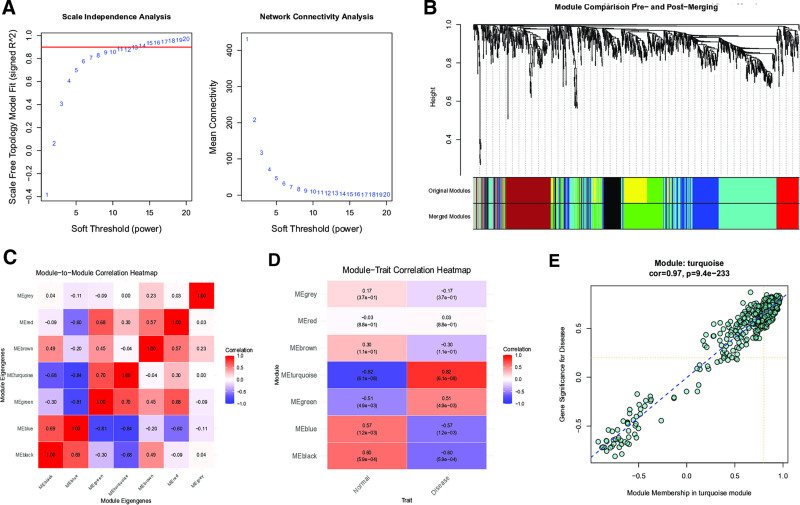
Identification of module genes.(A) Scale-free fit index of network topology was acquired through soft-thresholding power analysis.(B) Dendrogram of gene clustering results.(C) Heatmap showing correlations among different modules.(D) Heatmap depicting relationships between modules and clinical traits.(E) Correlation plot between gene members in the turquoise module and their gene significance values.

Through systematic WGCNA, we identified functionally relevant gene modules associated with disease progression. Following comprehensive evaluation of network topology, an optimal soft-thresholding power (β = 12) was selected to ensure scale-free network construction (Fig. 2A). Dynamic tree-cutting algorithms subsequently delineated 7 distinct co-expression modules (Fig. 2B), with hierarchical clustering demonstrating their interrelationships (Fig. 2C). Among these, the turquoise module exhibited the most significant biological relevance (Fig. 2D), showing a strong negative correlation with gene significance (*r* = −0.97, *P* = 9.4 × 10^−233^ Fig. 2E). This robust association prompted further investigation of the turquoise module, which comprised 376 candidate genes potentially involved in disease pathogenesis (Table S1, Supplemental Digital Content, https://links.lww.com/MD/R22).

### 3.2. Identifying the DEGs

**Figure 3.**
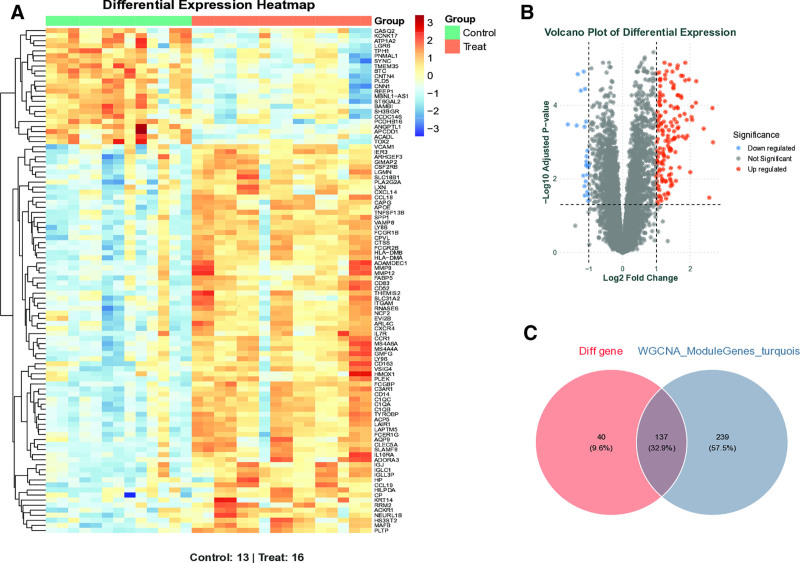
Identification of AS-associated DEGs; (A) volcano plot of differentially expressed genes. (B) Heatmap of DEGs. (C) Venn diagram of related differentially expressed genes. AS = atherosclerosis, DEGs = differentially expressed genes.

The expression profile dataset GSE28829 underwent rigorous normalization processing to ensure data comparability. Subsequent visualization through volcano plot (Fig. 3A) and heatmap (Fig. 3B) analyses revealed distinct gene expression patterns. Comparative analysis identified 238 DEGs that showed significant overlap with module genes (Fig. 3C), suggesting their potential functional relevance in disease pathogenesis.

### 3.3. Functional enrichment analysis of intersection genes

**Figure 4.**
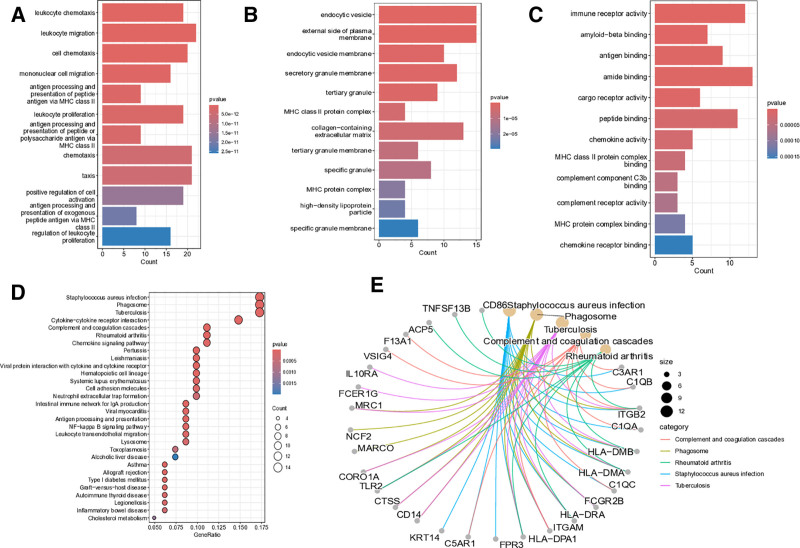
Functional enrichment analysis of DEGs; (A) GO biological processes; (B) GO cellular components; (C) GO molecular functions. (D) Bubble plot of KEGG pathways; (E) interaction diagram of KEGG pathways. DEGs = differentially expressed genes, GO = gene ontology, KEGG = kyoto encyclopedia of genes and genomes.

Gene ontology analysis demonstrated significant enrichment of the overlapping genes in key immunological processes. Biological process analysis identified 3 predominant functional clusters: leukocyte chemotaxis, leukocyte migration, and cellular chemotaxis (Fig. 4A), highlighting their critical role in immune cell trafficking. Cellular component mapping revealed strong associations with specialized membrane structures, including endocytic vesicles, the external plasma membrane surface, and secretory granule membranes (Fig. 4B). At the molecular level, these genes exhibited binding affinity for immune receptors, amyloid-beta proteins, and various amide compounds (Fig. 4C). Pathway enrichment analysis further established connections with clinically relevant infectious diseases, particularly Staphylococcus aureus infection and tuberculosis (Fig. 4D). Notably, the phagosome pathway emerged as a central hub for these gene products (Fig. 4E), suggesting their potential involvement in pathogen clearance mechanisms. These findings collectively underscore the immune regulatory functions of the identified gene set in host defense systems.

### 3.4. PPI network and hub genes analysis

**Figure 5.**
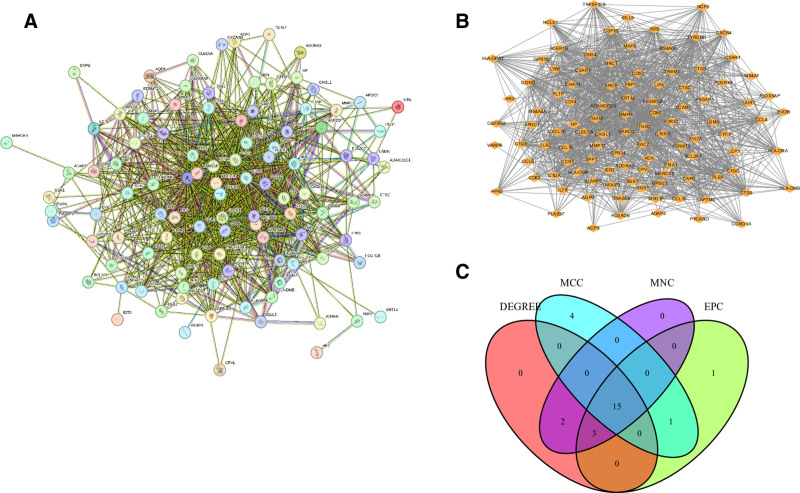
PPI network and Hub genes analysis. (A) The PPI network of the differentially expressed genes, where larger edge sizes indicate a higher degree. (B) The first module of the PPI network. (C) 14 common hub genes were identified by 4 algorithms of the cytoHubba plugin. PPI = protein–protein interactions

To elucidate functional relationships among the identified proteins, we constructed a PPI network using genes derived from the WGCNA analysis. The network was generated through STRING database analysis, yielding 132 interconnected nodes with 122 edges (Fig. 5A). Subsequent analysis using the MCODE plugin revealed densely connected network modules (Fig. 5B), suggesting potential functional complexes. For hub gene identification, we employed 4 distinct algorithms from the cytoHubba plugin, which converged on 15 consensus hub genes (Fig. 5C): TYROBP, CSF1R, ITGB2, CD163, FCER1G, ITGAM, CTSS, TLR2, CCR1, CD86, IRF8, C1QA, IL10RA, MRC1, and CCL4. These genes represent central players in the network topology and are likely crucial for module functionality.

### 3.5. Identification and validation of the key genes

**Figure 6.**
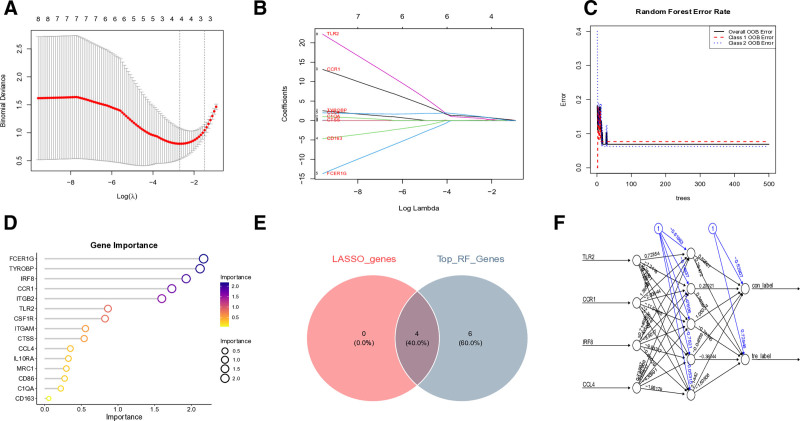
Identification of the key genes.(A and B) key genes were identified from hub genes using the machine learning LASSO regression method. (C and D) key genes were also identified from hub genes by the RF algorithm. (E) Four key genes were identified through overlap analysis: TLR2, CCR1, IRF8 and CCL4. (F) The ANN model of the key genes. ANN = artificial neural networks, LASSO = least absolute shrinkage and selection operator, RF = random forest.

**Figure 7.**
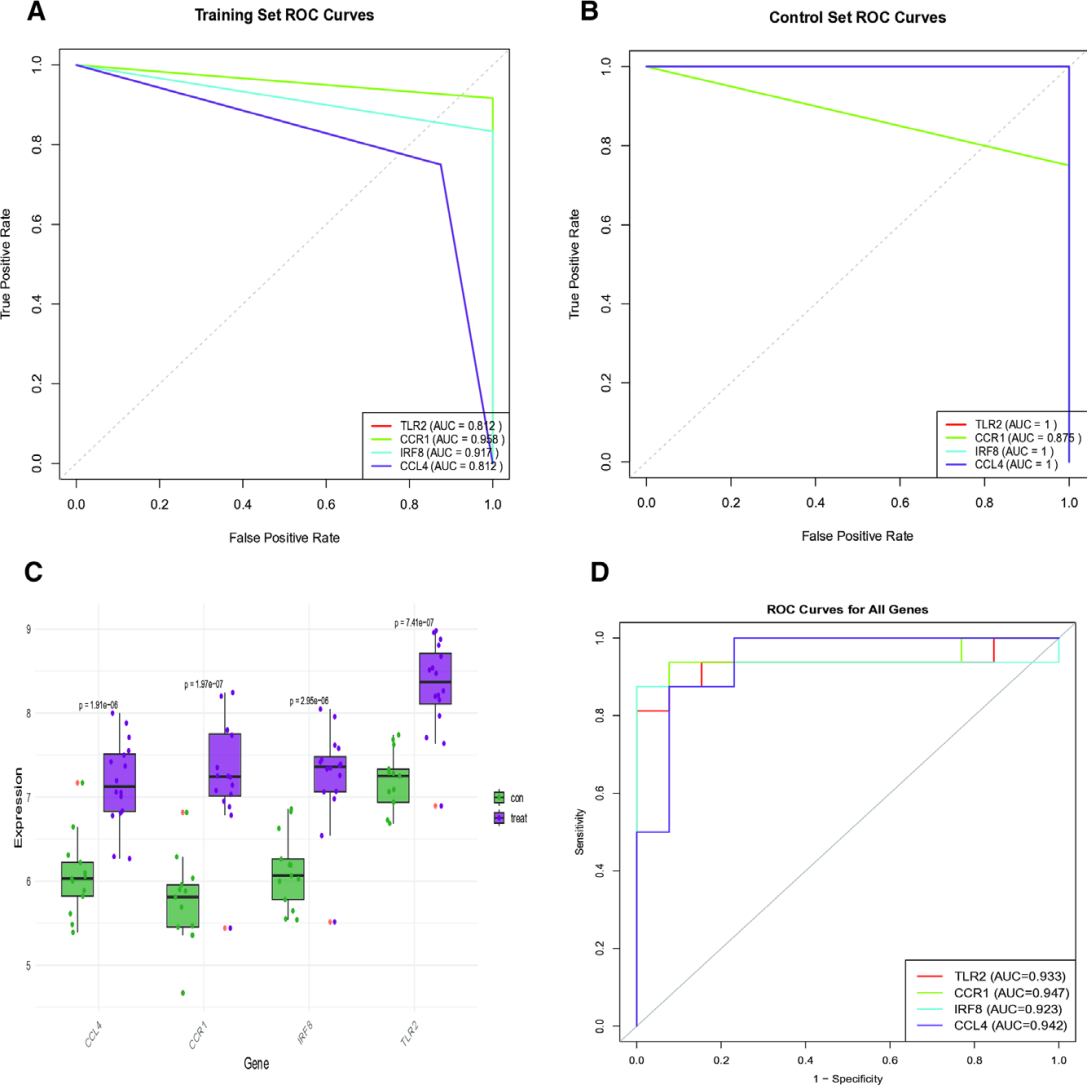
Validation of key genes. (A) ROC analysis was conducted to assess the performance of key genes within the experimental group of the ANN model.(B) ROC analysis was performed for key genes in the validation group of the ANN model to further evaluate their discriminatory power.(C) A notable finding revealed that the expression levels of these key genes were significantly reduced in AS.(D) ROC curves were generated to illustrate the diagnostic potential of key genes in AS. ANN = artificial neural network, AS = atherosclerosis, ROC = receiver operating characteristic.

**Figure 8.**
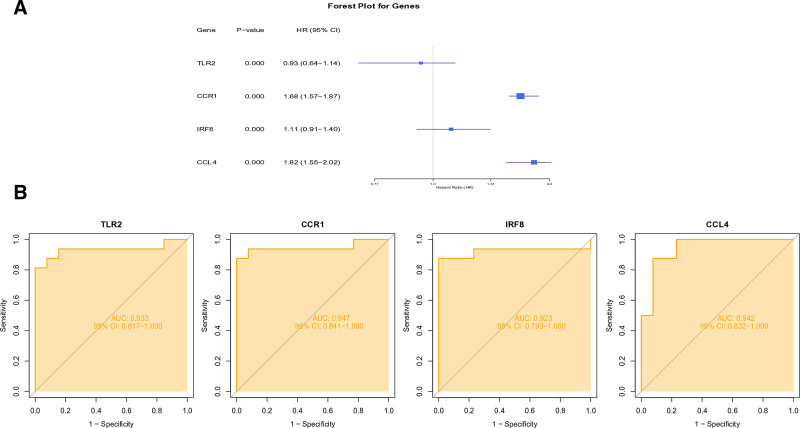
Visualization of key genes (A) Forest plot of key genes in AS; (B) ROC curves of key genes. AS = atherosclerosis.

Through integrated machine learning analysis, we identified 4 robust diagnostic biomarkers: toll like receptor 2 (TLR2), C-C motif chemokine receptor 1 (CCR1), interferon regulatory factor 8 (IRF8) and C-C motif chemokine ligand 4 (CCL4) (Fig. 6E). These biomarkers demonstrated consistent selection across both least absolute shrinkage and selection operator regression (Fig. 6A and B) and RF algorithms (Fig. 6C and D). The ANN diagnostic model incorporating these 4 genes exhibited outstanding performance characteristics (Fig. 6F). Specifically, the area under the curve values were 0.812 for TLR2, 0.958 for CCR1, 0.917 for IRF8, and 0.812 for CCL4 in the training cohort (Fig. 7A). Validation studies confirmed the diagnostic robustness of these biomarkers, with all maintaining AUC values above 0.8 in independent testing (Fig. 7B). Notably, expression profiling revealed significant differential expression patterns for TLR2, CCR1, IRF8 and CCL4 between patient samples and healthy controls (Fig. 7C and D). Clinical correlation analysis further characterized CCR1, IRF8 and CCL4 as disease risk factors, while identifying TLR2 as a protective factor (Fig. 8A). All 4 biomarkers achieved exceptional diagnostic accuracy with AUC values exceeding 0.9 in final evaluations (Fig. 8B).

### 3.6. Single-cell and immune infiltration analysis

**Figure 9.**
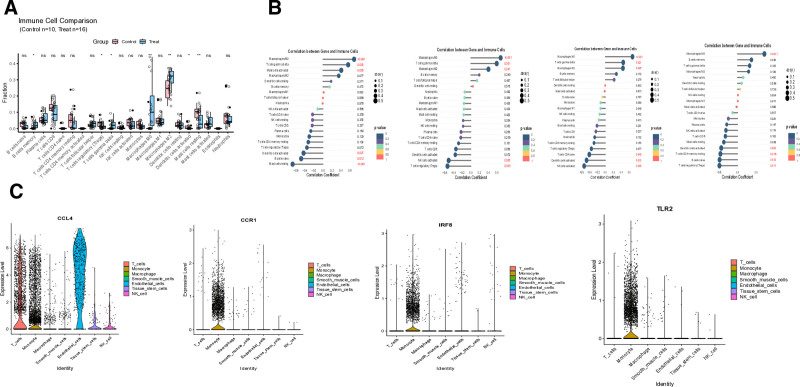
Immune infiltration and single-cell analysis.(A) Proportions of immune cells in AS patients and the control group. (B) Correlation between key genes and immune cells in AS patients. (C) Expression levels of key genes in various cell types of AS patients and the control group. AS = atherosclerosis.

Utilizing the CIBERSORT deconvolution algorithm, we characterized the immune landscape in AS patients compared to healthy controls. The analysis revealed significant alterations in specific immune cell populations (Fig. 9A): elevated infiltration of memory B cells, γδ T cells, M0 macrophages, and M2 macrophages, coupled with reduced levels of regulatory T cells (Tregs) and activated dendritic cells. Correlation analysis demonstrated important relationships between key biomarkers and immune cell subsets (Fig. 9B). The genes CCL4, CCR1 and IRF8 showed strong positive correlations with M0 macrophages, γδ T cells and M2 macrophage infiltration. Similarly, TLR2 expression significantly correlated with M0 macrophage abundance. To achieve single-cell resolution, we performed advanced scRNA-seq analysis incorporating rigorous quality control, normalization procedures, and unsupervised dimensionality reduction through clustering algorithms. This approach revealed that the 4 identified key genes exhibited predominant and elevated expression patterns within monocyte populations (Fig. 9C), suggesting their potential role in modulating monocyte function within the AS microenvironment.

### 3.7. Construction of an AS diagnostic model based on key genes

**Figure 10.**
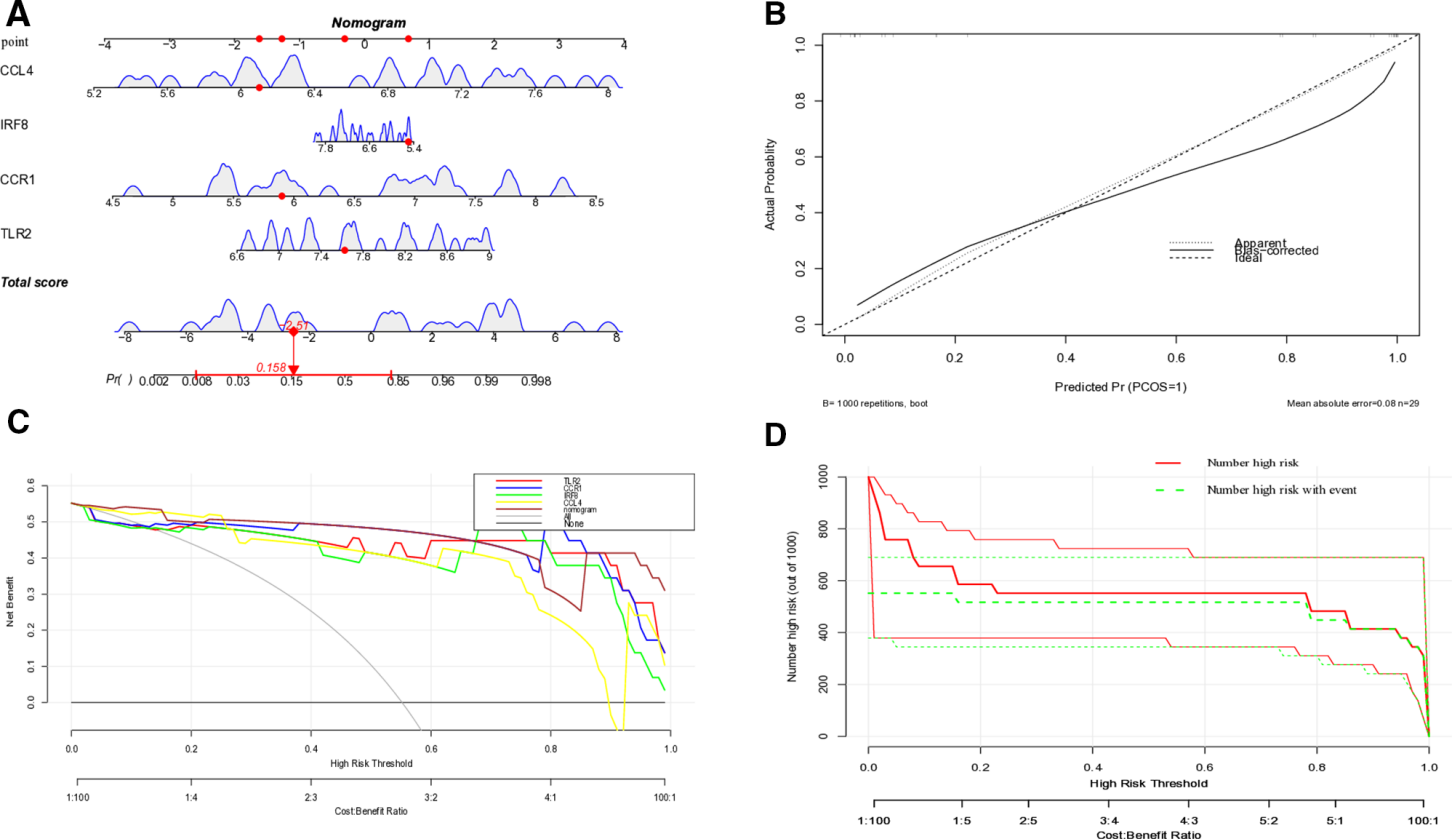
Construction and validation of the diagnostic model for AS; (A) nomogram for AS; (B) calibration curve of the model; (C) decision curve analysis; (D) clinical impact curve. AS = atherosclerosis.

We developed a comprehensive diagnostic nomogram incorporating 4 key biomarkers: TLR2, CCR1, IRF8, and CCL4. The model was implemented through an interactive dynamic nomogram interface (Fig. 10A), with performance evaluated using multiple validation approaches. Calibration analysis revealed strong concordance between predicted probabilities and observed outcomes (Fig. 10B), while decision curve analysis (Fig. 10C) demonstrated superior clinical utility across a wide range of risk thresholds (0.2–1.0). Importantly, the close alignment between predicted high-risk cases and actual event rates within this threshold range confirmed the model’s robust predictive capacity. These results collectively establish the nomogram as a reliable tool for AS risk stratification, with particular strength in identifying high-risk individuals (Fig. 10D).

### 3.8. Candidate drug prediction

**Figure 11.**
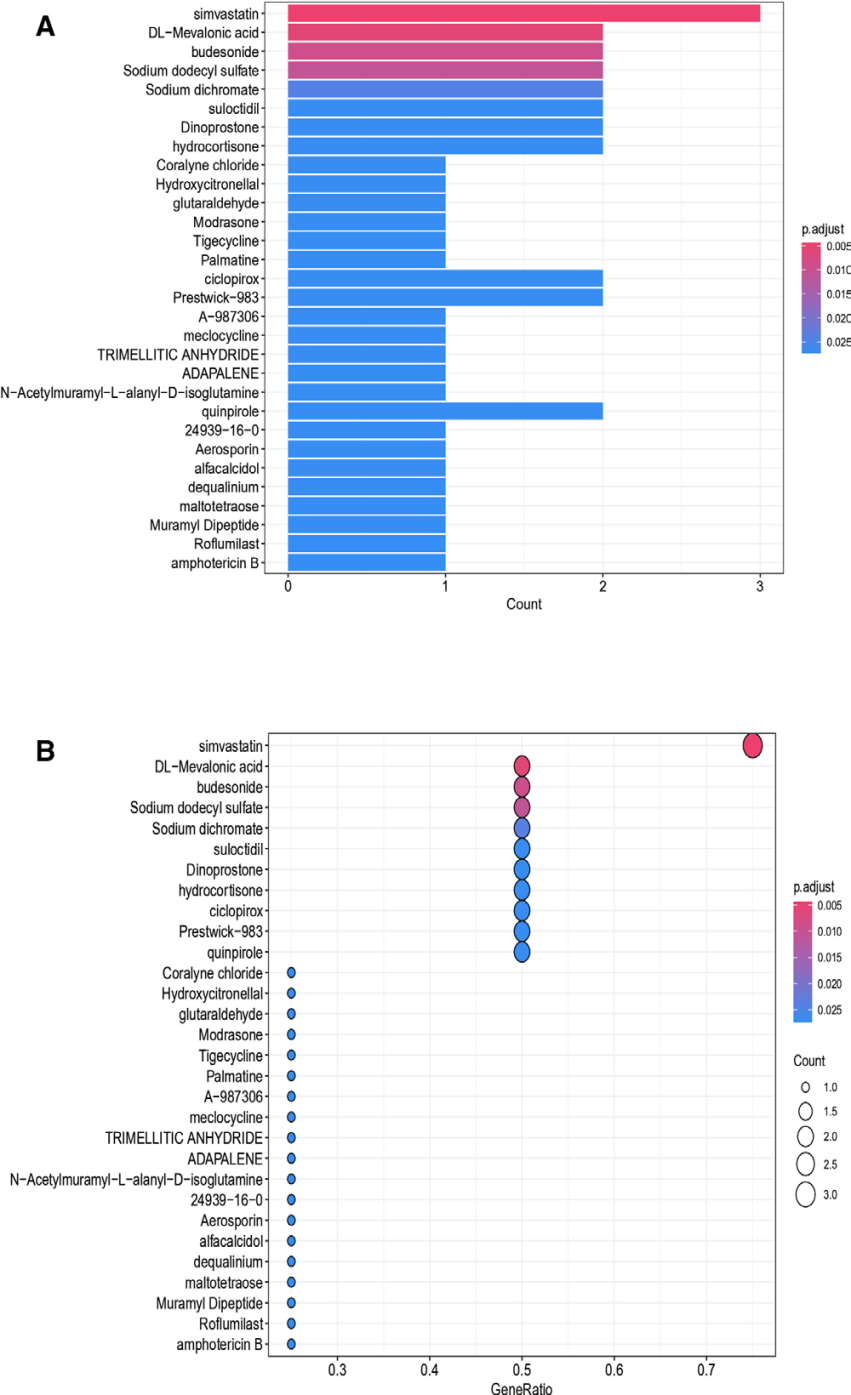
Visualization of drug enrichment of key genes.

Through systematic screening of the DSigDB database, we identified several pharmacologically active compounds showing significant associations with AS pathogenesis (adjusted *P*-value < 0.05). Our integrative computational approach revealed 3 particularly promising candidates: simvastatin (a HMG-CoA reductase inhibitor), mevalonic acid (a key cholesterol biosynthesis intermediate), and budesonide (a glucocorticoid receptor agonist). These compounds demonstrated the strongest statistical associations among all screened molecules (Fig. 11A and B). The identification of these FDA-approved compounds suggests their potential utility for AS intervention, as they may modulate disease-relevant pathways through known mechanisms of action. These findings provide a rational foundation for further preclinical evaluation and potential clinical translation in AS management.

### 3.9. Molecular docking

**Figure 12.**
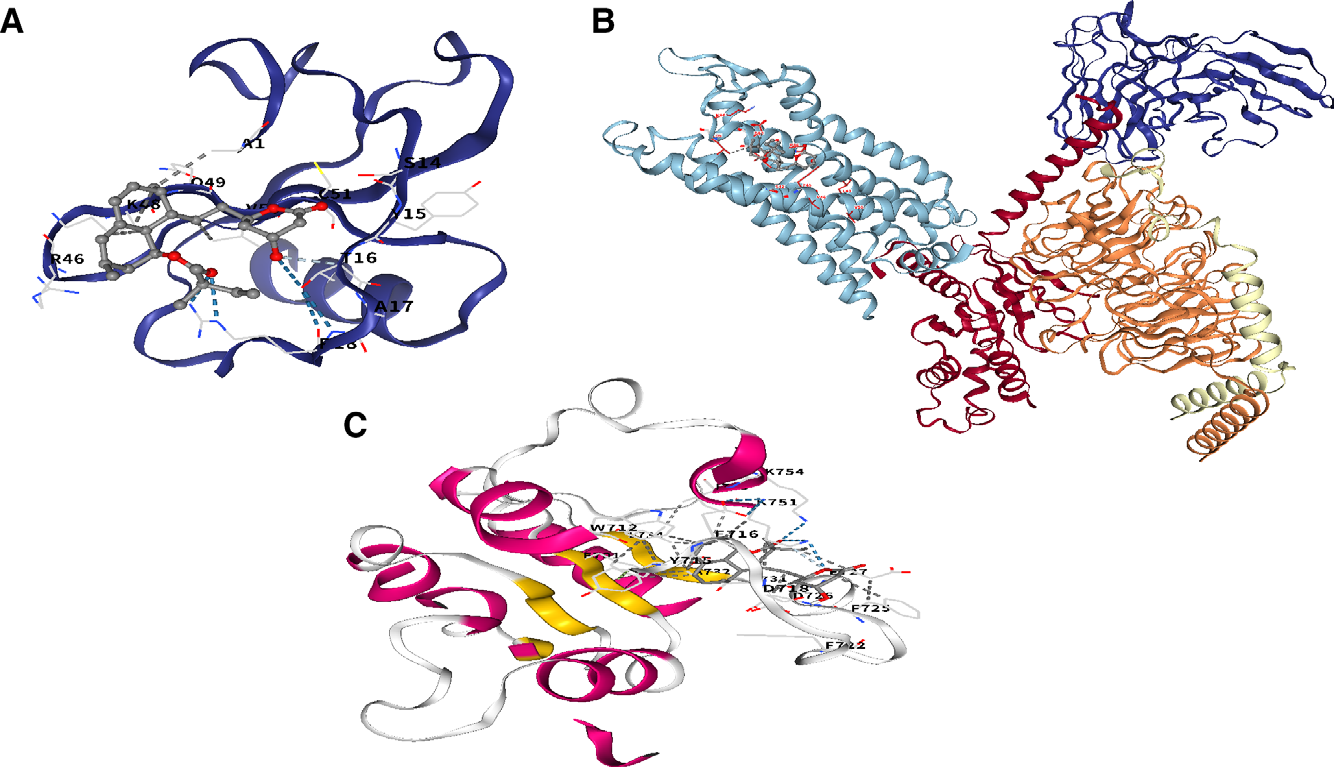
Schematic diagram of molecular docking.

To thoroughly evaluate the therapeutic potential of simvastatin in AS, this study employed molecular docking simulations to systematically analyze its binding affinities with key target proteins (TLR2, CCR1, and CCL4) involved in AS pathogenesis. The results demonstrated that simvastatin established stable interaction patterns with these proteins, evidenced by binding energies significantly below the conventional threshold (Fig. 12A–C). This indicates the formation of highly stable protein-ligand complexes, suggesting that simvastatin may modulate inflammation and vascular remodeling in AS through a multi-target mechanism.

## 4. Discussion

This study systematically identified key biomarkers and potential therapeutic targets for AS through WGCNA, DEG screening, functional enrichment analysis, and machine learning modeling. Four genes, TLR2, CCR1, IRF8, and CCL4, were found to exhibit significant differential expression in AS patients and were validated as core regulators of disease progression through multi-dimensional analyses, including PPI network construction, scRNA-seq and immune infiltration analysis. It is necessary to clarify the rationale for excluding other hub genes identified in the PPI network analysis. Initially, 15 consensus hub genes (TYROBP, CSF1R, ITGB2, CD163, FCER1G, ITGAM, CTSS, TLR2, CCR1, CD86, IRF8, C1QA, IL10RA, MRC1, CCL4) were screened using the cytoHubba plugin. Subsequently, 3 machine learning algorithms (LASSO regression, RF, and ANN) were integrated, and TLR2, CCR1, IRF8, and CCL4 were ultimately selected as core biomarkers. The remaining 11 hub genes were excluded based on 2 key considerations. From the perspective of diagnostic efficacy and stability, the diagnostic performance of these 4 genes is less affected by fluctuations in data distribution, and their stability is significantly higher than that of the other hub genes. By excluding the remaining 11 hub genes, subsequent studies will focus on TLR2, CCR1, IRF8, and CCL4, which ensures the reliability and translational potential of the research findings.

Single-cell sequencing revealed predominant expression of these 4 genes in monocyte populations, while CIBERSORT analysis demonstrated increased infiltration of γδ T cells, M0 macrophages, and M2 macrophages in AS patients, positively correlated with CCR1, IRF8, and CCL4 expression. These results suggest a critical role for the monocyte-macrophage-γδ T cell axis in shaping the inflammatory microenvironment of AS,^[28,29]^ operating in concert with TLR2-mediated innate immune activation. As a pattern recognition receptor, TLR2 recognizes both pathogen-associated molecular patterns (e.g, bacterial lipoproteins) and endogenous danger signals.^[30,31]^ In our study, we observed that TLR2 expression was upregulated in patients with AS, and this upregulation showed a positive correlation with M0 macrophage infiltration. This suggests that during the progression of AS, TLR2 may drive the activation of NF-κB-mediated immune defense responses in the early stage of chronic inflammation, promoting the recruitment and activation of macrophages.^[32,33]^ Notably, molecular docking simulations revealed that simvastatin exhibits a strong binding affinity to TLR2, indicating that simvastatin can competitively inhibit the interaction between TLR2 and its ligands. This mechanism may downregulate the excessive activation of TLR2 signaling, thereby alleviating the excessive inflammatory response driven by TLR2 in AS. These findings are consistent with the multi-faceted anti-inflammatory effects of statins, which extend beyond their classical lipid-lowering functions. The CCR1-CCL4 (MIP-1β) chemokine axis drives recruitment of monocytes and T cells to the vascular wall.^[34,35]^ Elevated CCR1 and CCL4 expression in AS was significantly associated with infiltration of γδ T cells and M2 macrophages, which secrete pro-fibrotic cytokines (e.g., IL-17, TGF-β) to promote vascular smooth muscle cell proliferation and plaque fibrosis.^[36,37]^ Given the proven anti-inflammatory efficacy of CCR1 inhibitors in rheumatoid arthritis, this study provides cross-disease mechanistic evidence for their potential use in AS.^[38,39]^ Molecular docking further revealed that simvastatin forms stable complexes with CCR1 and CCL4 via hydrogen bonding and hydrophobic interactions, potentially blocking chemokine-receptor binding and inhibiting immune cell migration.IRF8, a key transcription factor driving monocyte differentiation into pro-inflammatory M1 macrophages, was positively correlated with M0 macrophage infiltration and enriched in phagosome pathways, suggesting its role in modulating plaque stability through enhanced clearance of apoptotic cells.^[40,41]^ However, excessive IRF8 activation may also promote pro-inflammatory cytokine secretion, exacerbating vascular inflammation. This dual functionality positions IRF8 as a promising precision therapy target, though the mechanism by which simvastatin interacts with IRF8 requires experimental validation. A diagnostic nomogram incorporating the 4 biomarkers demonstrated excellent performance (AUC > 0.9) in both training and validation cohorts, with calibration and decision curve analyses confirming its clinical utility for early risk stratification, particularly in high-risk subgroups (e.g, patients with diabetes or smoking history). Compared to traditional imaging techniques (e.g, CTA), biomarker-based assays offer minimally invasive, dynamic monitoring of plaque biological activity, a critical unmet need in AS diagnosis.

Molecular docking confirmed that simvastatin forms stable complexes with TLR2, CCR1, and CCL4, potentially exerting multi-target effects in AS: beyond inhibiting HMG-CoA reductase for lipid lowering, simvastatin may suppress NF-κB-mediated inflammation via TLR2,^[42,43]^ disrupt immune cell recruitment through the CCR1-CCL4 axis, and reduce oxidative stress.^[44,45]^ As an FDA-approved drug with established safety in cardiovascular disease, simvastatin represents a promising candidate for repurposing in AS. Additional candidates from DSigDB, including mevalonic acid and budesonide, warrant further investigation.

The compelling diagnostic performance (AUC > 0.9) of our 4-gene biomarker nomogram prompts consideration of its practical application in clinical settings. The transition from a statistical model to a clinically viable tool hinges on its seamless integration into existing diagnostic pathways. We envision a synergistic role for our nomogram alongside current standards. Specifically, the expression levels of TLR2, CCR1, IRF8, and CCL4 could be quantified via minimally invasive methods such as quantitative PCR or enzyme-linked immunosorbent assays (ELISA) using peripheral blood samples. This “liquid biopsy” approach offers a significant advantage by providing a dynamic snapshot of the systemic immune-inflammatory activity driving AS, which is not captured by static anatomical imaging. In a proposed integrated workflow, the biomarker nomogram could serve as an initial risk stratification or prescreening tool. For instance, in primary care or high-risk clinics (e.g., for patients with diabetes or smoking history), a blood draw could generate a nomogram-based risk score. Individuals identified as “high-risk” by this model could then be prioritized for more costly and invasive confirmatory imaging tests, such as CTA or duplex ultrasonography. This 2-stage strategy could optimize healthcare resource utilization, reduce unnecessary imaging exposure, and facilitate earlier detection of biologically active AS before the onset of severe symptoms or complications. Furthermore, the model’s ability to reflect immune status positions it as a potential tool for monitoring therapy response, such as assessing the anti-inflammatory effects of statins like simvastatin, beyond their lipid-lowering efficacy.

By integrating multi-omics data and applying machine learning, this study identified TLR2, CCR1, IRF8, and CCL4 as novel biomarkers for AS, constructed a nomogram that can effectively predict AS risk, and confirmed that simvastatin may regulate immune inflammation and vascular remodeling through multiple targets, collectively providing a new strategy for AS diagnosis and treatment. Looking ahead, 2 directions of work can be advanced based on this research: on one hand, establishing a multi-omics database for AS that integrates multi-center clinical data (including risk factors, imaging findings, and prognosis) with multi-dimensional omics data, while unifying quality control standards and ensuring data security to lay a foundation for optimizing diagnostic models; on the other hand, developing a AS organoid model by constructing a 3D model using vascular-related cells differentiated from patient-induced pluripotent stem cells (iPSCs), which can be used to verify the function of key genes and screen candidate drugs, thereby providing a basis for subsequent experiments, further promoting the development of AS diagnosis and treatment technologies, and improving patient prognosis.

## 5. Limitations and future directions

Although this study has unveiled key disease mechanisms and potential intervention targets through multi-dimensional integrated analysis, several limitations warrant attention. First, the validation of biomarkers relied solely on public datasets, which might have bias,^[46]^ lacking verification in independent clinical cohorts, particularly regarding expression heterogeneity across different disease subtypes, which requires further confirmation. Second, while single-cell analysis identified monocytes as critical cell types, the functional distinctions between specific subpopulations remain undefined. Additionally, mechanistic investigations of candidate drugs were limited to molecular docking, necessitating validation of their efficacy and safety through cellular assays and animal models. Future research may focus on the following directions:1) Developing multi-omics databases incorporating clinical and pathological features to optimize diagnostic models; 2) Utilizing organoids or gene-edited animal models to dissect the dynamic roles of key genes in immune-tissue interactions.

## 6. Conclusion

In summary, our study identifies TLR2, CCR1, IRF8, and CCL4 as core biomarkers of AS, enabling an immune-based diagnostic nomogram. This shifts the paradigm from anatomical assessment to dynamic risk profiling. Coupled with the multi-target potential of simvastatin, our work paves the way for mechanistically targeted strategies in AS management.

## Acknowledgments

We extend our heartfelt gratitude for the wealth of resources provided by the public database, which has offered invaluable support to our work and research.

## Author contributions

**Funding acquisition:** Jian Shi.

**Investigation:** Han Li, Jian Shi.

**Software:** Jian Shi.

**Writing – original draft:** Wangwei Zhou.

**Writing – review & editing:** Min Huang, Han Li, Jian Shi.

## Supplementary Material


